# Oscillating Glucose Induces the Increase in Inflammatory Stress through Ninjurin-1 Up-Regulation and Stimulation of Transport Proteins in Human Endothelial Cells

**DOI:** 10.3390/biom13040626

**Published:** 2023-03-30

**Authors:** Laura Toma, Gabriela M. Sanda, Camelia S. Stancu, Loredan S. Niculescu, Mina Raileanu, Anca V. Sima

**Affiliations:** Lipidomics Department, Institute of Cellular Biology and Pathology “Nicolae Simionescu” of the Romanian Academy, 8, B.P. Hasdeu Street, 050568 Bucharest, Romania; laura.toma@icbp.ro (L.T.); gabriela.sanda@icbp.ro (G.M.S.); camelia.stancu@icbp.ro (C.S.S.); loredan.niculescu@icbp.ro (L.S.N.); mina.raileanu@nipne.ro (M.R.)

**Keywords:** endothelial cell dysfunction, inflammatory stress, oscillating glucose, oxidative stress, Ninjurin-1, transendothelial transport proteins

## Abstract

Clinical data implicate fluctuations of high levels of plasma glucose in cardiovascular diseases. Endothelial cells (EC) are the first cells of the vessel wall exposed to them. Our aim was to evaluate the effects of oscillating glucose (OG) on EC function and to decipher new molecular mechanisms involved. Cultured human ECs (EA.hy926 line and primary cells) were exposed to OG (5/25 mM alternatively at 3 h), constant HG (25 mM) or physiological concentration (5 mM, NG) for 72 h. Markers of inflammation (Ninj-1, MCP-1, RAGE, TNFR1, NF-kB, and p38 MAPK), oxidative stress (ROS, VPO1, and HO-1), and transendothelial transport proteins (SR-BI, caveolin-1, and VAMP-3) were assessed. Inhibitors of ROS (NAC), NF-kB (Bay 11-7085), and Ninj-1 silencing were used to identify the mechanisms of OG-induced EC dysfunction. The results revealed that OG determined an increased expression of Ninj-1, MCP-1, RAGE, TNFR1, SR-B1, and VAMP-3 andstimulated monocyte adhesion. All of these effects were induced bymechanisms involving ROS production or NF-kB activation. *NINJ-1* silencing inhibited the upregulation of caveolin-1 and VAMP-3 induced by OG in EC. In conclusion, OG induces increased inflammatory stress, ROS production, and NF-kB activation and stimulates transendothelial transport. To this end, we propose a novel mechanism linking Ninj-1 up-regulation to increased expression of transendothelial transport proteins.

## 1. Introduction

Fluctuation of glucose concentration is a phenomenon that occurs daily (postprandial) in human blood. Intraday glucose fluctuations measured mainly as variations in the amplitude of glycemic excursions are more important in type 2 diabetes mellitus (T2DM) patients than in normal subjects [1]. Glucose fluctuations were associated with blood pressure variability in normotensive, normoglycemic subjects [2], with an increased risk for major adverse cardiovascular events and all-cause mortality in patients with no history of diabetes or cardiovascular diseases (CVD) [3] and with excess hospital complications and mortality in critically ill patients without diabetes [4]. In diabetic patients, recent studies report that blood glucose fluctuations are associated with oxidative and inflammatory stress, generating micro- and macrovascular complication characteristics for diabetes [5,6,7,8,9]. Ongoing studies indicate that in diabetic patients, glucose fluctuation is associated with increased arterial stiffness [10], left ventricular remodeling [11], accelerated progression of coronary atherosclerosis [12], risk of major adverse coronary events including myocardial infarction and stroke [13,14] and increased risk of mortality [13,15]. Recently, in type 2 diabetic patients, the postprandial plasma glucose oscillations were associated with the modification of the lipid profile towards an atherogenic one [16]. Regarding the microvascular complications, it was shown that glucose fluctuations lead to chronic kidney disease [17], diabetic retinopathy [18], or diabetic neuropathy [19] in type 1 diabetic patients [8]. All these complications are the consequences of the deleterious effects of glucose oscillations on different cell types, including: pancreatic islet beta cells [20,21,22], cardiomyocytes [23,24], neurons [25], renal and endothelial cells [8,26]. Due to serious health problems generated by glucose fluctuations, continuous glucose monitoring systems are increasingly used for diabetic patients to help them regulate their lifestyle and adjust their medication [8,27,28]. Different non-pharmacological or pharmacological strategies to reduce glucose variations in the human plasma were proposed. Among these, low carbohydrate diet, exercise training, and weight reduction have been recommended to alleviate the glucose fluctuations in vivo [29,30,31,32,33]. Although glucagon-like peptide-1 analogues and dipeptidyl-peptidase 4 inhibitors show a significant impact on glucose variability (GV) [33,34], and combined therapies seem to be more efficient, there are still issues that need to be addressed in order to reduce the harmful effects of glucose variations in diabetic patients [35,36]. Endothelial cells (ECs) are the first cells in contact with each alteration in the blood. In normal conditions, ECs are important contributors to vascular homeostasis by controlling the traffic of inflammatory cells and regulating the vascular tone, the transport of macromolecules, and the fibrinolytic processes. In pathological conditions, EC become dysfunctional, suffering a shift towards a pro-inflammatory, vasoconstrictor and pro-thrombotic phenotype [37]. The increase in the transendothelial transport process (transcytosis) is another characteristic of the dysfunctional EC observed in CVD [37].

Oscillating glucose (OG) is a term used in the literature to depict the fluctuation of glucose concentration that can appear daily in the plasma of normal or diabetic subjects. A growing body of evidence demonstrates that OG determines EC dysfunction (ECD), manifested as decreased nitric oxide (NO) bioavailability, increased oxidative stress, senescence, and apoptosis [38,39,40]. Interestingly, some studies report that these effects are more pronounced in the presence of OG than in that of constant high glucose (HG), suggesting that OG may have a higher impact than HG in the generation of the cardiovascular complications that appear in diabetes [38,39,41]. However, the mechanisms of OG-induced ECD are not completely elucidated. Data regarding the effect of OG on the expression of inflammatory proteins that play an important role in CVD progression such as TNFα receptor 1 (TNFR1) or the receptor for advanced glycation end products (RAGE) are lacking. Moreover, the effect of OG on proteins involved in transendothelial transport (transcytosis), such as scavenger receptor BI (SR-BI), caveolin-1 (Cav-1), and vesicle-associated membrane protein 3 (VAMP-3), are not reported. Ninjurin-1 (Ninj-1) is a protein present on the surface of EC. It was recently reported to participate in the progression of different pathologies, including diabetes and CVD [42,43,44]. The involvement of Ninj-1 in these pathologies can be explained by the fact that this protein mediates important cellular processes, such as the generation of inflammatory and oxidative stress, apoptosis [42,45], regulation of NO synthesis [46], or membrane rupture during lytic cell death [47]. Data regarding the effect of OG on Ninj-1 expression are also missing.

The aim of the present study was to identify new molecular mechanisms regarding the deleterious effects of OG-exposure of ECs to define novel therapeutic targets that could help to restore EC proper function (essential for the homeostasis of the vascular system) exposed to glucose fluctuations. An experimental model that mimics EC exposure to short-term diurnal postprandial blood glucose oscillations was designed and used. This was conducted by exposing cultured human ECs alternatively to HG or normal glucose (NG) every 3 h during daytime, for 72 h, and to NG during night-time, in an attempt to reproduce a normal program of meals, in contrast to other experimental models that have used glucose oscillations during larger time pulses (of 6 h, 12 h, or even 24 h) [38,41,48]. The effects of OG compared to constant HG and NG on inflammatory and oxidative stress, on proteins controlling transcytosis, and on the endoplasmic reticulum stress (ERS) were evaluated. To deepen the understanding of the molecular mechanisms, specific inhibitors for reactive oxygen species (ROS) and pro-inflammatory nuclear factor kB (NF-kB) as well as Ninj-1 silencing were used to highlight new possible therapeutic targets to ameliorate OG-induced ECD.

## 2. Materials and Methods

### 2.1. Reagents

Dulbecco’s Modified Eagle’s Medium (DMEM), streptomycin, penicillin, neomycin, d-glucose, l-glucose, mannitol, 2’,7’-dichlorofluorescein diacetate (DCFH-DA), 2′7′-bis (2-carboxyethyl)-5(6)-carboxyfluorescein acetoxymethyl ester (BCECF-AM), protease inhibitor cocktail, sodium orthovanadate and sodium fluoride, N-acetyl cysteine (NAC) and Bay11-7085 (Bay) were from Sigma-Aldrich Co., St. Louis, MO, USA. Antibodies to human glucose regulated protein 78 (GRP78, sc-58774), phospho-eukaryotic Initiation Factor 2α (p-eIF2α, sc-101670), total eIF2α (t-eiF2α, sc-11386), p65 nuclear factor-kB (NF-kB) subunit (sc-372), lamin B1 (sc-377000), phospho-p38 mitogen-activated protein kinase (MAPK) (p-p38, sc-17852-R), total p38 MAPK (t-p38, sc-7149), Ninj-1 (sc-136295), TNFR1 (sc-8436), VAMP-3 (sc-514843), Cav-1 (sc-53564), heme oxygenase 1 (HO-1, sc-390991) and β-actin (sc-47778) were from Santa Cruz Biotechnology, Santa Cruz, CA, USA. *NINJ-1* siRNA (siNinj, sc-75915), control scramble siRNA (Scr, sc-37007) and siRNA Transfection Reagent (sc-29528) were from Santa Cruz Biotechnology, Santa Cruz, CA, USA, and Oligofectamine™ Transfection Reagent (12252011) was from Invitrogen, Thermo Fisher Scientific, Waltham, MA, USA. p22phox (ab75941), monocyte chemoattractant protein-1 (MCP-1, ab9669), RAGE (ab547410) and SR-BI (ab396) antibodies were from Abcam, UK, and fetal calf serum (FCS) was from Euroclone, EU. TRIzol reagent, HighCapacity cDNA Reverse Transcription Kit, and SyBr Select Master Mix were from Applied Biosystems, Foster City, CA, USA.

### 2.2. Cell Culture and Experimental Design

**Figure 1:**
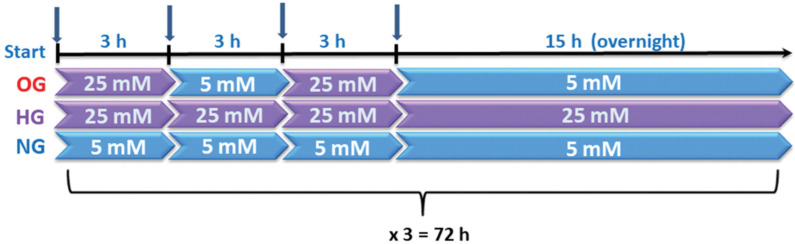
Graphical representation of the experimental design. Cells were exposed to 5 mM glucose (NG), 25 mM glucose (HG) or alternatively to 5 mM/25 mM glucose (OG) at every 3 h, for 72 h.

Human umbilical vein endothelial cells (EA.hy926 line) commercialized by ATCC (Manassas, VA, USA) were cultured in 5 mM glucose DMEM supplemented with 10% FCS in the presence of antibiotics (streptomycin/penicillin and neomycin) for 5 days. At confluency, cell media were changed at every 3 h in DMEM containing alternatively 5 mM glucose and 25 mM glucose (OG condition), in the presence of 5% FCS, for 48 h. The cells from OG group were kept into 5 mM glucose overnight, to mimic the in vivo situation. In addition, cells incubated in DMEM containing 5 mM glucose (NG) or 25 mM glucose (HG) exchanged at every 3 h were used. After 48 h, the cells were deprived by FCS and exposed for another 24 h to NG/HG/OG, using the same protocol as above (Figure 1).

For osmotic control, the cells from EA.hy926 line were incubated with L-glucose or mannitol using the same protocol for OG. To investigate the mechanisms by which OG induce EC dysfunction, in some experiments, NAC (7.5 mM) or Bay (15 µM) were added to the OG experimental condition, for the whole period of incubation.

Primary HUVECs commercially available from ATCC (Manassas, VA, USA) were also used in some experiments. Primary cells were grown as manufacturers recommend. When cells reached 70–80% confluency, the incubation protocol presented above was used.

The experimental OG model was chosen to mimick the post-prandial hyperglycaemia in diabetic patients, as well as aged normo-glycemic subjects. The chosen concentrations of glucose correspond to normal glucose levels (5 mM = 90 mg/dL) or to diabetic postprandial values of glucose (25 mM = 450 mg/dL).

### 2.3. In Vitro Silencing of Ninjurin-1 in OG-Exposed EC

*NINJ-1* gene expression was silenced in EC from EAhy.926 cell line (50–80% confluency) by using 50 nM specific siRNA for *NINJ-1* and the recommended transfection reagent (Santa Cruz Biotechnology, Santa Cruz, CA, USA) or 0.4% oligofectamine, according to manufacturer instructions. The transfection of EC primary cells was performed using specific siRNA for *NINJ-1* (25 nM) and INTERFERin^®^ transfection reagent from Polyplus (Polyplus, Illkirch-Graffenstaden, France, EU), according to the manufacturer’s instructions. Twenty-four hours after the transfection, ECs were incubated in OG conditions as described above. After 72 h of incubation, EC were harvested, lysed, and processed for Western Blot analysis. For comparison, ECs exposed to OG or ECStransfected with the same concentration of scrambled, irrelevant siRNA and exposed to OG were used.

### 2.4. RNA Isolation and Gene Expression Measurement

After the experimental procedure, the samples were lysed in TRIzol reagent and immediately stored at −80 °C. On the next day, the total RNA was isolated according to manufacturer instructions for TRIzol method, and the obtained nucleic acid concentrations were quantified spectrophotometrically using NanodropLite (Thermo Fisher Scientific, Waltham, MA, USA). ARN purity was estimated by evaluating the A260/280 ratio. An amount of 1 μg of total RNA was reverse transcribed using the HighCapacity cDNA Reverse Transcription Kit in a reaction containing 50 U MultiScribe Reverse Transcriptase enzyme and random primers. The reverse transcription reaction was conducted in a Verity Thermal Cycler (Applied Biosystems, Foster City, CA, USA) using the program optimized by the manufacturers (25 °C, 10 min; 37 °C, 120 min; 85 °C, 5 min; 4 °C hold). The obtained cDNA was kept at −20 °C until further processing. For cDNA amplification, a SyBr Select Master Mix and 150 nM of specific primers for *NINJ-1, MCP-1, RAGE/AGER, TNFR1/TNFRSF1A*, tumour necrosis factor α (*TNFα*), *SR-BI/SCARB, CAV-1, VAMP-3*, vascular peroxidase 1/peroxidasin (*VPO1/PXDN*), *p22phox/CYBA*, *GRP78*, spliced X-box protein 1 (*sXBP1*), and β-*actin* (*ACTB*, as housekeeping gene) were used. A volume of 2 µL of cDNA was amplified in a ViiA7 Real-Time PCR system, assisted by the Quant Studio Real-Time PCR software (Applied Biosystems, Foster City, CA, USA), in 10 µL of final reaction volume, using the recommendations from SyBr Select Master Mix assay. The SyBr Select Master Mix assay contained all the necessary ingredients for cDNA optimal amplification (SYBR™ GreenER™ dye, AmpliTaq™ DNA Polymerase, dNTPs blend, heat-labile UDG, ROX passive reference dye, and optimized buffer components). The amplification program was as follows: (1) a holding stage containing two steps, one of 50 °C, 2 min and another of 95 °C, 2 min for AmpliTaq DNA Polymerase activation; (2) a stage of amplification containing 40 cycles of denaturation at 95 °C, 15 s and annealing at 60 °C, 1 min—with fluorescence reading, and (3) a melting curve stage: 95 °C, 15 sec; 60 °C, 1 min and continuous reading of SyBr Green fluorescence (0.05 °C increment) until 95 °C. The sequence accession number, specific sequence of each primer and amplicon length are given in detail in Table S1 from Electronic Supplementary Materials. Amplification of the samples was conducted in technological duplicate. The negative controls for amplification were probes with RNA-se free water instead of cDNA, and the results showed no amplification into these probes. The specificity of the PCR reaction was demonstrated by the appearance of a single peak in the melting curve. The Ct for β-actin did not vary significantly between the experimental conditions. The relative quantification of the amplification products was performed using 2(-Delta Delta C(T)) method, according to [49], versus NG that was attributed to value 1.

### 2.5. Quantification of Protein Expression in Cell Lysates

After the incubation, ECs were washed two times with cold phosphate-buffered saline (PBS), and total cellular lysates were obtained using the RadioImmuno Precipitation Assay (RIPA) buffer enriched with protease inhibitors (Sigma cocktail) and phosphatase inhibitors (1 mM sodium fluoride and 2 mM sodium orthovanadate). In addition, for the analysis of NF-kB nuclear translocation, fractions enriched in nuclear proteins were obtained as previously described [50]. In total, 30–50 μg total cell protein or 25 μg nuclear lysates were separated on 10–12% SDS-polyacrylamide gel electrophoresis (SDS-PAGE), transferred to nitrocellulose membranes, and processed as in [51]. The relative protein expression (protein of interest normalized to β-actin) was determined by the densitometric analysis of the digital image using TotalLab 100 software (Sigma-Aldrich Co., St. Louis, MO, USA), and expressed relative to NG or OG (in the experiments where inhibitors were used).

### 2.6. Measurement of Secreted MCP-1 in the Cell Culture Medium

Secreted MCP-1 level was determined in the culture media collected after the exposure of ECs to OG, HG or NG, as detailed. Cells’ culture media were slightly centrifuged to eliminate the cellular debris, concentrated, and processed as in [52]. The level of the secreted MCP-1 was normalized to total cell protein and expressed relative to NG or OG (in the experiments where inhibitors were used).

### 2.7. Monocyte Adhesion Assay

After the experimental procedure, monocyte adhesion to from EA.hy926 line was measured as previously reported [51]. In brief, human monocyte from THP-1 cell line (ATCC, Manassas, VA, USA) were loaded by incubation with 10 μmol/L BCECF-AM fluorochrome for 30 min, at 37 °C. Fluorescently-labelled THP-1 were added and left to interact at 37 °C with ECs previously exposed to OG/HG/NG. After 30 min, the non-adhered THP-1 were removed by gentle washing with DMEM, while the adhered THP-1 were lysed in 1% Triton X-100 in 0.1 M NaOH solution. The fluorescence of BCECF was measured at 485 nm (excitation)/535 nm (emission), using a spectrofluorometer Tecan Infinite M200 (Tecan, Austria). The adhesion of monocytes to ECs was presented as fold of NG or OG in the experiments where inhibitors were used.

### 2.8. Intracellular ROS Level Measurements

Intracellular ROS were evaluated in EC from EA.hy926 line by using the cell-permeant DCFH-DA fluorochrome as described in [51]. The fluorescence emitted by ROS-sensitive fluorophore, was detected at 485 nm/535 nm using the spectrofluorometer Tecan Infinite M200 (Tecan, Grödig, Austria). ROS levels were expressed as relative fluorescence units per microgram of total cell protein and presented as fold change of NG.

### 2.9. Statistical Analysis

SPSS software (IBM SPSS, IBM Ireland, Dublin, Ireland) was used for the statistical analysis of the data. One-way ANOVA was employed to evaluate the deleterious effects of OG exposure comparing three experimental groups (the NG-, HG-, and OG- exposed cells). Two post-hoc tests, Tukey HSD test (honestly significant difference) or Tamhane test, depending on the homogeneity of variances (evaluated by Levene test), were used to compare the data groups two by two (OG vs. NG, HG vs. NG, or OG vs. HG). The Mann-Whitney (U-test) was used for the validation of the obtained results. Statistically significant were considered *p* < 0.05 values. All data were expressed as mean ± standard deviation (SD) and are representative for at least three independent experiments.

## 3. Results

### 3.1. Oscillating Glucose Up-Regulates Endothelial Expression of Ninj-1 and MCP-1, Promoting Increased Adhesion of Monocytes to ECs from EA.hy.926 Cell Line

**Figure 2:**
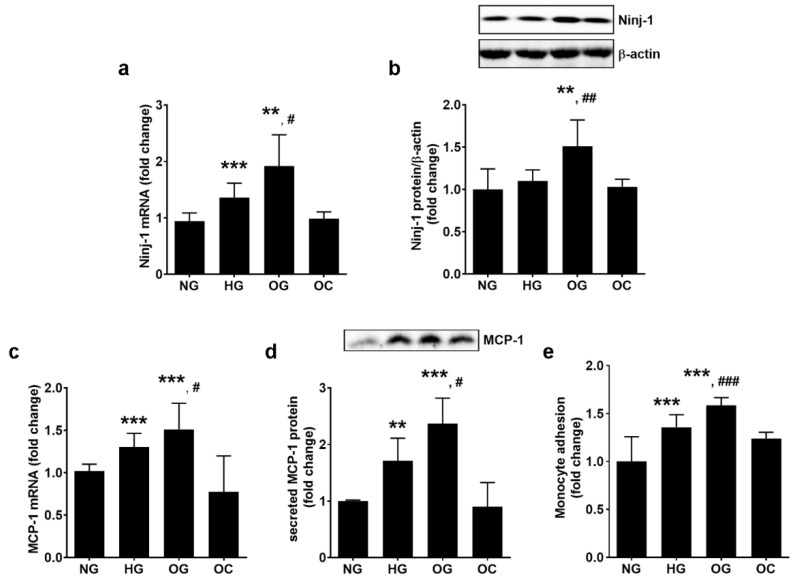
Oscillating glucose stimulates Ninj-1, MCP-1, and monocyte adhesion to ECs to a greater extent than HG. Cells were exposed to 5 mM glucose (NG), 25 mM glucose (HG) or alternatively to 5 mM/25 mM glucose (OG) at every 3 h, for 72 h. Cells exposed alternatively to L-glucose or mannitol (using the same protocol as for OG) was considered osmotic control (OC). (**a**,**c**) mRNA levels of Ninj-1 (**a**) and MCP-1 (**c**); (**b**) Ninj-1 protein expression in whole cell lysate relative to β-actin (representative blots and densitometric analysis); (**d**) secreted MCP-1 in the culture media relative to total cell protein (representative blots and densitometric analysis); (**e**) monocyte adhesion to endothelial cells. All data are expressed as fold change versus NG and presented as mean ± SD. ** *p* < 0.01, *** *p* < 0.001 vs. NG; ^#^ *p* < 0.05, ^##^ *p* < 0.01, ^###^ *p* < 0.001 vs. HG.

The effects of OG exposure on EC inflammatory stress were evaluated by assessing the gene and protein expression of the adhesion molecule Ninj-1 and monocyte chemoattractant protein 1 (MCP-1). The results showed that OG determined a statistically significant up-regulation of *NINJ-1* gene (1.90 ± 0.65 for OG, 1.36 ± 0.26 for HG, and 1.00 ± 0.13 for NG; *p* < 0.01 for OG vs. NG and *p* < 0.05 for OG vs. HG; *p* < 0.001 for all studied groups by ANOVA test) and protein expression (1.51 ± 0.31 for OG, 1.10 ± 0.13 for HG, and 1.00 ± 0.24 for NG; *p* < 0.01 for both OG vs. NG and OG vs. HG; *p* < 0.001 for all studied groups by ANOVA test) (Figure 2a,b).

OG determined the highest up-regulation of *MCP-1* gene expression (1.51 ± 0.31 for OG, 1.30 ± 0.16 for HG, and 1.00 ± 0.07 for NG; *p* < 0.001 for OG vs. NG and *p* < 0.05 for OG vs. HG; *p* < 0.001 for all studied groups by ANOVA test) and of the secreted protein (2.51 ± 0.31 for OG, 1.86 ± 0.16 for HG, and 1.00 ± 0.02 for NG; *p* < 0.001 for OG vs. NG and *p* < 0.05 for OG vs. HG; *p* < 0.001 for all studied groups by ANOVA) (Figure 2c,d).

To validate these results, a functional test for monocyte adhesion to ECs exposed to OG, HG or, NG was performed. The results showed that OG stimulated the adhesion of monocytes to ECs to a greater extent than HG (1.58 ± 0.08 for OG, 1.36 ± 0.13 for HG, and 1.00 ± 0.26 for NG; *p* < 0.001 for both OG vs. NG and OG vs. HG; *p* < 0.001 for all studied groups by ANOVA) (Figure 2e).

### 3.2. Oscillating Glucose Up-Regulates the Expression of TNFR1 and RAGE in ECs from EA.hy926 Cell Line

The gene expression of *TNFα* was measured by real-time PCR, and the expression of pro-inflammatory receptors TNFR1 and RAGE were evaluated by real-time PCR and Western blot in the whole EC lysates.

**Figure 3:**
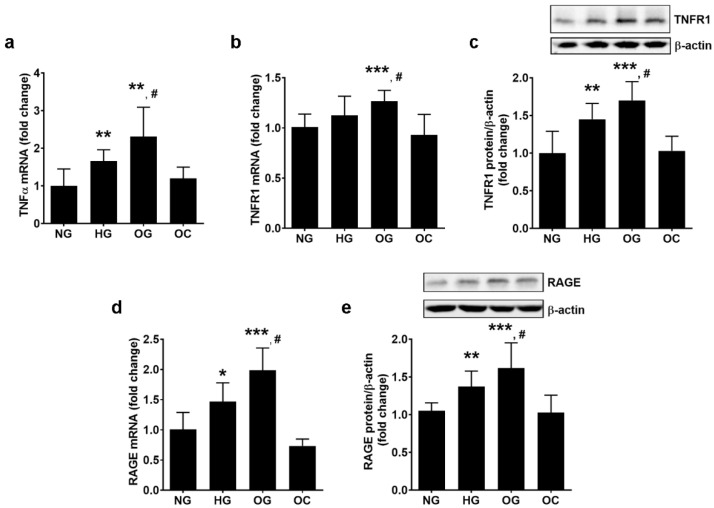
Oscillating glucose stimulates the expression of TNFα, TNFR1 and RAGE in ECs to a greater extent than constant high glucose. Cells were exposed to 5 mM glucose (NG), 25 mM glucose (HG) or alternatively to 5 mM/25 mM glucose (OG) at every 3 h, for 72 h. Cells exposed alternatively to L-glucose or mannitol (using the same protocol as for OG) was considered osmotic control (OC). (**a**,**b**,**d**) mRNA levels of TNFα (**a**), TNFR1 (**b**) and RAGE (**d**); (**c**,**e**) protein expression of TNFR1 (**c**) and RAGE (**e**) in EC lysates relative to β-actin (representative blot and densitometric analysis. All data are expressed as fold change versus NG and presented as mean ± SD. * *p* < 0.05, ** *p* < 0.01,*** *p* < 0.001 vs. NG; ^#^ *p* < 0.05 vs. HG.

The results indicated that OG increases the gene expression of *TNFα* to a greater extent than HG (2.31 ± 0.78 for OG, 1.66 ± 0.3 for HG, and 1.00 ± 0.45 for NG; *p* < 0.01 for OG vs. NG and *p* < 0.05 for OG vs. HG; *p* < 0.001 for all studied groups by ANOVA, as shown in Figure 3a, in agreement with other published data [39,43].

OG determined the up-regulation of *TNFR1* gene expression to a greater extent than HG (1.27 ± 0.11 for OG, 1.12 ± 0.19 for HG and 1.00 ± 0.13 for NG, *p* < 0.001 for OG vs. NG and *p* < 0.05 for OG vs. HG; *p* < 0.001 for all studied groups by ANOVA). Correspondingly, OG stimulated TNFR1 protein expression to a greater extent than HG (1.70 ± 0.25 for OG, 1.44 ± 0.21 for HG, and 1.00 ± 0.37 for NG; *p* < 0.001 for OG vs. NG and *p* < 0.05 for OG vs. HG; *p* < 0.001 for all studied groups by ANOVA, as shown in Figure 3b,c).

OG has stimulated *RAGE* gene expression to a greater extent than HG (1.98 ± 0.37 for OG, 1.47 ± 0.31 for HG, and 1.00 ± 0.29 for NG; *p* < 0.001 for OG vs. NG and *p* < 0.05 for OG vs. HG; *p* < 0.001 for all studied groups by ANOVA) and RAGE protein expression (1.62 ± 0.33 for OG, 1.37 ± 0.20 for HG, and 1.00 ± 0.30 for NG; *p* < 0.001 for OG vs. NG and *p* < 0.05 for OG vs. HG; *p* < 0.001 for all studied groups by ANOVA, as shown in Figure 3d,e).

### 3.3. Oscillating Glucose and Constant High Glucose Stimulate the Proteins Involved in the Regulation of Transendothelial Transport

**Figure 4:**
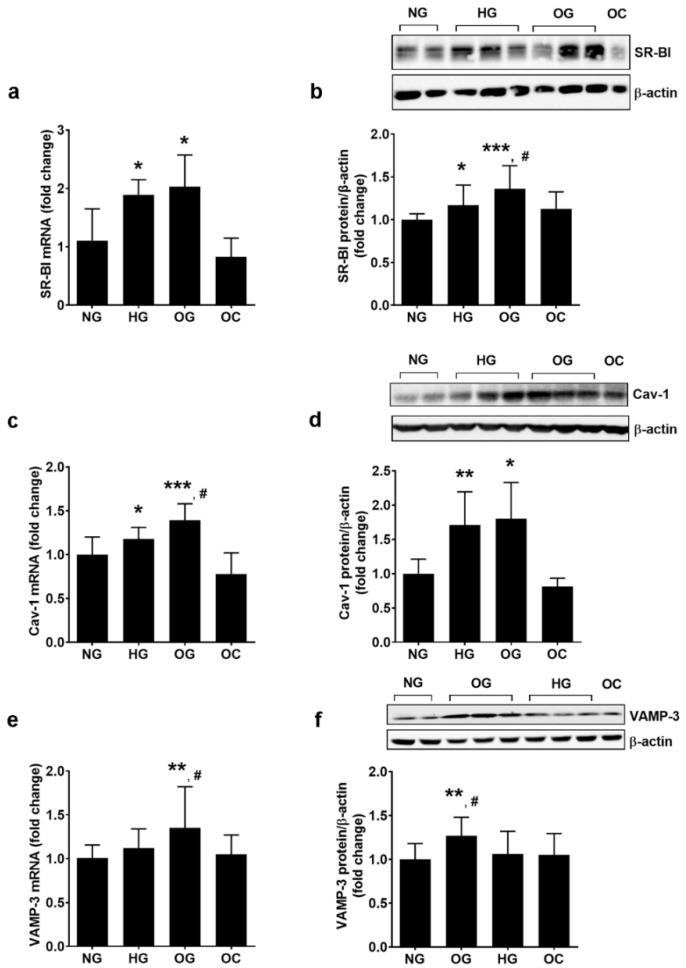
Oscillating glucose alters the expression of proteins involved in transendothelial transport of molecules. Cells were exposed to 5 mM glucose (NG), 25 mM glucose (HG) or alternatively to 5 mM/25 mM glucose (OG) at every 3 h, for 72 h. Cells exposed alternatively to l-glucose or mannitol (using the same protocol as for OG) was considered osmotic control (OC). (**a**,**c**,**e**) mRNA levels of SR-BI (**a**), Cav-1 (**c**) and VAMP-3 (**e**); (**b**,**d**,**f**) protein expression of SR-BI (**b**), Cav-1 (**d**) and VAMP-3 (**f**) in EC lysates relative to β-actin (representative blot and densitometric analysis). All data are expressed as fold change versus NG and presented as mean ± SD. * *p* < 0.05, ** *p* < 0.01, *** *p* < 0.001 vs. NG; ^#^ *p* < 0.05 vs. HG.

EC plays an important role in the homeostasis of the vascular wall due to their function as a semipermeable barrier. The effect of OG on SR-BI, Cav-1 and VAMP-3, proteins involved in the transendothelial transport of molecules was evaluated in EC total lysate by real-time PCR and Western Blot. The exposure of ECs to OG or HG determined the increase in *SR-BI* gene (2.03 ± 0.54 for OG, 1.89 ± 0.26 for HG, and 1.00 ± 0.54 for NG; *p* < 0.05 for OG or HG vs. NG, *p* = ns for OG vs. HG; *p* < 0.001 for all studied groups by ANOVA) and protein expression (1.37 ± 0.27 for OG, 1.14 ± 0.21 for HG, and 1.00 ± 0.08 for NG; *p* < 0.001 for OG vs. NG and *p* < 0.05 for OG vs. HG; *p* < 0.001 for all studied groups by ANOVA) (Figure 4a,b).

*CAV-1* gene expression was up-regulated by OG and HG (1.39 ± 0.19 for OG, 1.18 ± 0.13 for HG, and 1.00 ± 0.20 for NG; *p* < 0.001 for OG vs. NG and *p* < 0.05 for OG vs. HG; *p* < 0.001 for all studied groups by ANOVA), and the protein expression was stimulated similarly by both conditions (1.60 ± 0.50 for OG, 1.46 ± 0.32 for HG, and 1.00 ± 0.15 for NG; *p* < 0.05 for OG vs. NG, *p* = ns for OG vs. HG; *p* < 0.05 for all studied groups by ANOVA) (Figure 4c,d).

*VAMP-3* gene expression was stimulated more by OG as compared to NG (1.36 ± 0.47 for OG, 1.12 ± 0.22 for HG, and 1.00 ± 0.12 for NG; *p* < 0.01 for OG vs. NG; *p* < 0.05 for all studied groups by ANOVA). OG determined a statistically significant up-regulation of VAMP-3 protein compared to HG or NG (1.27 ± 0.26 for OG, 1.06 ± 0.21 for HG, and 1.00 ± 0.23 for NG; *p* < 0.01 for OG vs. NG, *p* < 0.05 for OG vs. HG; *p* < 0.05 for all studied groups by ANOVA, as shown in Figure 4e,f).

### 3.4. Oscillating Glucose Determines Increased ROS Production and Endoplasmic Reticulum Stress in ECs from EA.hy926 Cell Line

**Figure 5:**
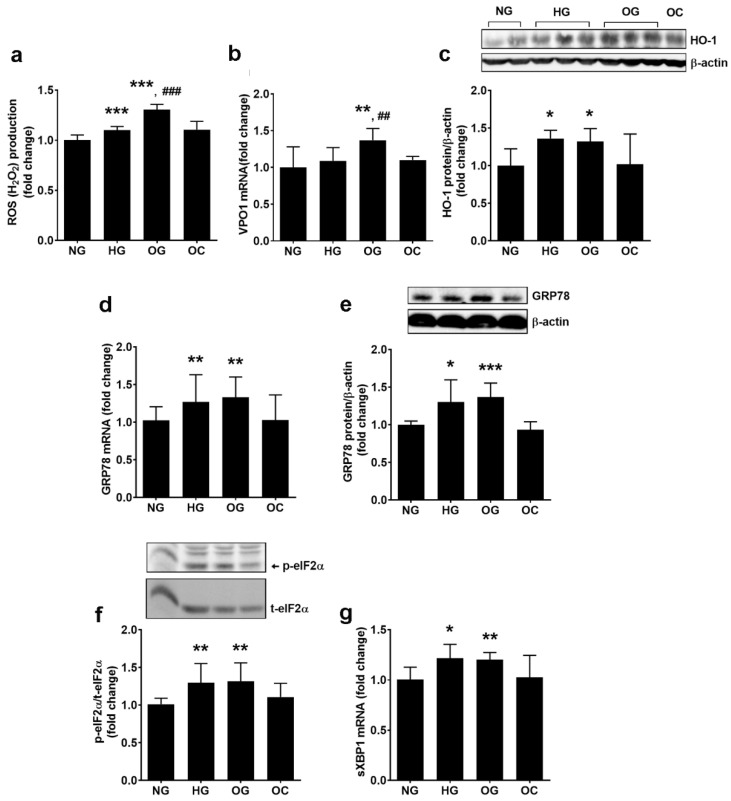
Oscillating glucose generates oxidative stress and endoplasmic reticulum stress in EC. Cells were exposed to 5 mM glucose (NG), 25 mM glucose (HG) or alternatively to 5 mM/25 mM glucose (OG) at every 3 h, for 72 h. Cells exposed alternatively to l-glucose or mannitol (using the same protocol as for OG) were considered osmotic control (OC). (**a**) Intracellular reactive oxygen species (ROS) levels; (**b**,**d**,**g**) mRNA levels of VPO1 (**b**), GRP78 (**d**) and spliced XBP1 (sXBP1) (**g**); (**c**,**e**) protein expression of HO-1 (**c**) GRP78 (**e**); (**f**) phosphorylated eIF2α relative to total eIF2α (*p*-eIF2α/t-eIF2α) (representative blot and densitometric analysis). All data are expressed as fold change versus NG and presented as mean ± SD. * *p* < 0.05, ** *p* < 0.01, *** *p* < 0.001 vs. NG; ^##^ *p* < 0.01, ^###^ *p* < 0.001 vs. HG.

It is well known that ROS play an important role in promoting ECD; thus, the effect of OG-exposure on intracellular ROS level was evaluated. The results showed that OG significantly increased the intracellular ROS levels to a greater extent than HG (1.30 ± 0.05 for OG, 1.10 ± 0.04 for HG, and 1.00 ± 0.05 for NG; *p* < 0.001 for both OG vs. NG and OG vs. HG; *p* < 0.001 for all studied groups by ANOVA, as shown in Figure 5a).

To investigate the ROS sources stimulated by our experimental conditions, the expression of VPO1, p22phox (the regulatory subunit of NADPH oxidase, NADPHox) and of HO-1 was evaluated in ECs exposed to OG, HG, and NG. The results showed that OG stimulated *VPO1/PXDN* gene expression to a greater extent than HG or NG (1.37 ± 0.16 for OG, 1.09 ± 0.18 for HG, and 1.00 ± 0.28 for NG; *p* < 0.01 for OG vs. NG and HG, *p* = ns for HG vs. NG, *p* < 0.01 for all studied groups by ANOVA, as shown in Figure 5b). Data showed that OG stimulated *p22phox* gene expression more compared with HG (1.66 ± 0.25 for OG, 1.23 ± 0.29 for HG, and 1.00 ± 0.21 for NG; *p* < 0.001 for OG vs. NG and *p* < 0.05 for OG vs. HG; *p* < 0.01 for all studied groups by ANOVA). OG increased p22phox protein expression by 53% and HG by 16% compared to NG (1.53 ± 0.19 for OG, 1.16 ± 0.25 for HG, and 1.00 ± 0.11 for NG; *p* < 0.01 for OG vs. NG and *p* < 0.05 for OG vs. HG; *p* < 0.01 for all studied groups by ANOVA, as shown in Figure S1a,b). HO-1 protein expression was similarly increased by both HG and OG (1.32 ± 0.17 for OG, 1.36 ± 0.10 for HG vs. 1.00 ± 0.22 for NG; *p* < 0.05 for OG or HG vs. NG, as shown in Figure 5c).

The functionality of the endoplasmic reticulum in OG-exposed ECs was investigated by assessing the intracellular levels of the following ER stress markers: glucose regulated protein 78 (GRP78), phosphorylated eIF2α, and spliced X box protein 1 (sXBP1). Exposure of ECs to OG determined the increase in *GRP78* gene (1.36 ± 0.22 for OG and 1.00 ± 0.16 for NG; *p* < 0.01 for OG vs. NG; *p* < 0.01 for all studied groups by ANOVA) and protein expression (1.37 ± 0.18 for OG and 1.00 ± 0.05 for NG; *p* < 0.001 for OG vs. NG; *p* < 0.05 for all studied groups by ANOVA), the increase in eIF2α phosphorylation (1.32 ± 0.24 for OG and 1.00 ± 0.07 for NG; *p* < 0.01 for OG vs. NG; *p* < 0.001 for all studied groups by ANOVA), and the stimulation of *XBP1* mRNA splicing (1.20 ± 0.07 for OG and 1.00 ± 0.12 for NG; *p* < 0.01 for OG vs. NG; *p* < 0.05 for all studied groups by ANOVA), as shown in Figure 5d–g. HG stimulated ERS sensors similarly to OG, no statistically significant differences being quantified between the two experimental conditions (Figure 5d–g).

### 3.5. Oscillating Glucose Activates the Pro-Inflammatory NF-kB Transcription Factor and p38 MAPK

**Figure 6:**
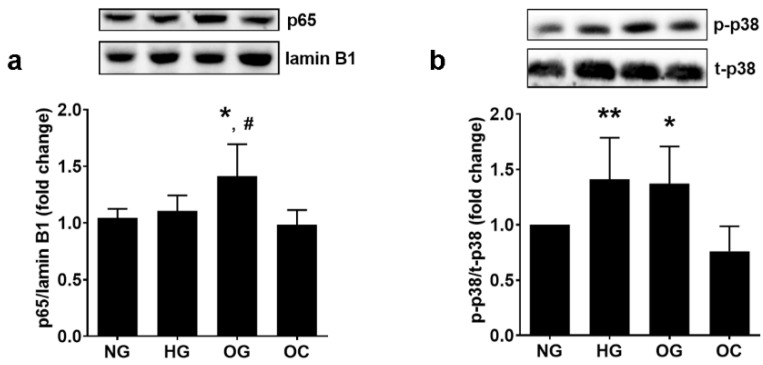
Oscillating glucose stimulates pro-inflammatory NF-kB and p38 MAPK in EC. Cells were exposed to 5 mM glucose (NG), 25 mM glucose (HG) or alternatively to 5 mM/25 mM glucose (OG) at every 3 h, for 72 h. Cells exposed alternatively to L-glucose or mannitol (using the same protocol as for OG) were considered osmotic control (OC). (**a**) Nuclear p65 NF-kB subunit relative to lamin B1 (p65/lamin B1); (**b**) the ratio of phosphorylated p38 MAPK relative to total p38 MAPK (p-p38/t-p38). All data are expressed as fold change versus NG and presented as mean ± SD. * *p* < 0.05, ** *p* < 0.01 vs. NG; ^#^ *p* < 0.05 vs. HG.

Oxidative stress and ERS are strong inducers of the pro-inflammatory signaling pathways initiated by NF-kB and p38 MAPK. Thus, the translocation of NF-kB into the nucleus and the phosphorylation of p38 MAPK were evaluated in ECs from EA.hy926 line in our experimental conditions. The results indicated that OG determined a higher translocation of NF-kB into the nucleus compared to NG or HG (1.41 ± 0.28 for OG, 1.11 ± 0.14 for HG and 1.00 ± 0.08 for NG; *p* < 0.05 for both OG vs. NG and OG vs. HG; *p* < 0.01 for all studied groups by ANOVA), as shown in Figure 6a.

The phosphorylation level of p38 MAPK in ECs was increased by OG to a similar levelwith HG, as compared with cells incubated in NG (1.37 ± 0.23 for OG, 1.41 ± 0.37 for HG, and 1.00 ± 0.00 for NG; *p* < 0.05 for OG vs. NG and *p* = ns for OG vs. HG; *p* < 0.01 for all studied groups by ANOVA), as shown in Figure 6b.

### 3.6. Oscillating Glucose Stimulates Inflammatory Stress by Inducing Oxidative Stress and NF-kB Transcription Factor in ECs from EA.hy926 Line

**Figure 7:**
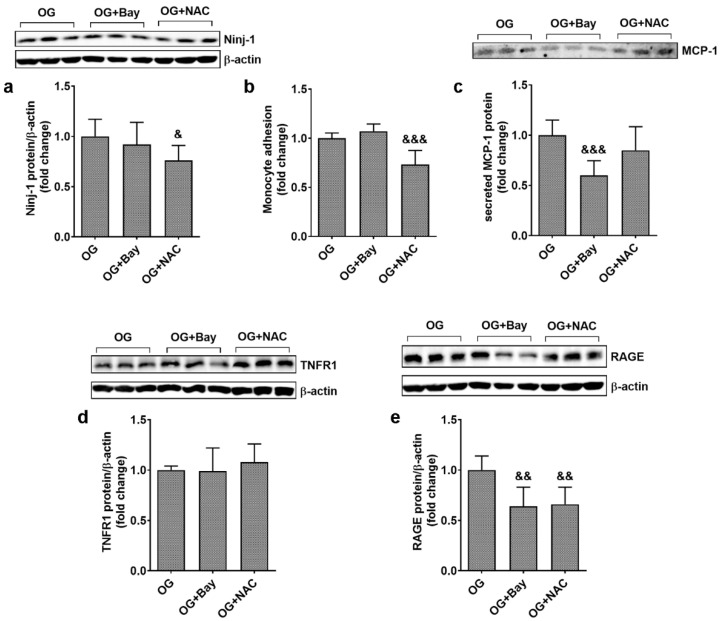
Inhibitors for oxidative stress and NF-kB reduce inflammation in ECs exposed to oscillating glucose. Cells were exposed alternatively to 5 mM/25 mM glucose (OG) at every 3 h, for 72 h. N-acetyl-cysteine (NAC, 7.5 mM) or Bay11-7085 (Bay, 15 µM) were added to the OG experimental condition, for the whole period of incubation. (**a**,**d**,**e**) Protein expression of Ninj-1 (**a**), TNFR1 (**d**) and RAGE (**e**) in EC lysates relative to β-actin (representative blot and densitometric analysis); (**b**) monocyte adhesion to endothelial cells; (**c**) secreted MCP-1 in the culture media relative to total cell protein (representative blots and densitometric analysis). All data are expressed as fold change versus OG and presented as mean ± SD. ^&^ *p* < 0.05, ^&&^ *p* < 0.01, ^&&&^ *p* < 0.001 vs. OG.

To evaluate the mechanisms by which OG induces inflammatory stress, ECs from EA.hy926 line was exposed to OG in the presence/absence of inhibitors for NF-kB, Bay11-7085 (Bay), or large spectrum antioxidant N-acetyl cysteine (NAC), and the protein expression of pro-inflammatory stress markers were evaluated by Western Blot. The results showed that NAC reduced significantly Ninj-1 protein expression (0.76 ± 0.15 for NAC vs. 1.00 ± 0.17; *p* < 0.05), while Bay had no effect (0.92 ± 0.22 for protein, *p* = ns). Accordingly, monocyte adhesion was significantly reduced by NAC (0.73 ± 0.14 for NAC, 1.07 ± 0.07 for Bay, and 1.00 ± 0.05 for OG; *p* < 0.001 for NAC vs. OG and *p* = ns for Bay vs. OG), shown in Figure 7a,b. MCP-1 protein expression was not influenced by NAC but was decreased by Bay in ECs exposed to OG (0.59 ± 0.15 for Bay; 0.85 ± 0.23 for NAC vs. 1.00 ± 0.13 for OG; *p* < 0.001 for Bay vs. OG), shown in Figure 7c.

Regarding the mechanism of regulation of pro-inflammatory receptors, our results show that neither Bay nor NAC affects TNFR1 protein expression (0.99 ± 0.23 for Bay and 1.08 ± 0.18 for NAC vs. 1.00 ± 0.04 for protein expression, *p* = ns), as shown in Figure 7d. In contrast, statistically significant decreases in RAGE gene expression were observed with both inhibitors (0.64 ± 0.20 for Bay and 0.66 ± 0.17 for NAC vs. 1.00 ± 0.15 for OG; *p* < 0.01 for Bay or NAC vs. OG), as shown in Figure 7e.

### 3.7. Oxidative Stress and NF-kB Modulate the Expression of Proteins Involved in Transendothelial Transport 

**Figure 8:**
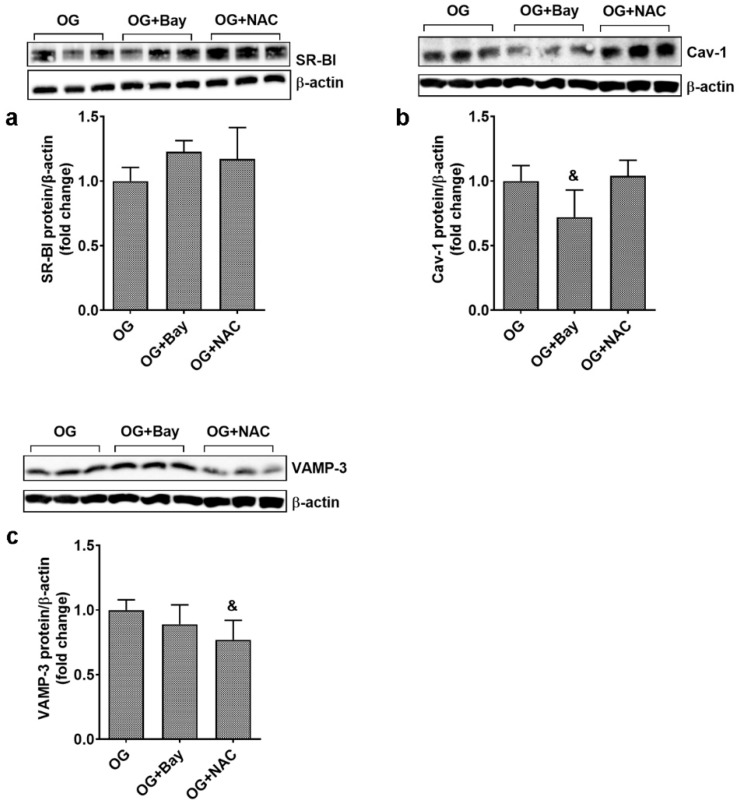
Inhibitors for oxidative stress and NF-kB reduce the expression of Cav-1 and VAMP-3 in ECs exposed to oscillating glucose. Cells were exposed alternatively to 5 mM/25 mM glucose (OG) at every 3 h, for 72 h. N-acetyl-cysteine (NAC, 7.5 mM) or Bay11-7085 (Bay, 15 µM) were added to the OG experimental condition, for the whole period of incubation. (**a**–**c**) Protein expression of SR-BI (**a**), Cav-1 (**b**) and VAMP-3 (**c**) in EC lysates relative to β-actin (representative blot and densitometric analysis). All data are expressed as fold change versus OG and presented as mean ± SD. ^&^ *p* < 0.05 vs. OG.

The effect of Bay and NAC was also tested on the expression of SR-BI, Cav-1, and VAMP-3 in ECs from EA.hy926 line exposed to OG. Results showed that Bay and NAC have no effect on SR-BI protein expression (1.22 ± 0.08 for Bay and 1.17 ± 0.24 for NAC, vs. 1.00 ± 0.10 for OG), while Cav-1 protein expression was significantly reduced by Bay (0.72 ± 0.21 for Bay and 1.04 ± 0.12 for NAC vs. 1.00 ± 0.12 for OG; *p* < 0.05 for Bay and *p* = ns for NAC). Regarding VAMP-3, this protein expression was statistically reduced by NAC (0.77 ± 0.15 for NAC vs. 1.00 ± 0.08, *p* < 0.05), as shown in Figure 8a–c.

### 3.8. Ninjurin 1 Silencing Decreases the Expression of Proteins Involved in Transendothelial Transport

**Figure 9:**
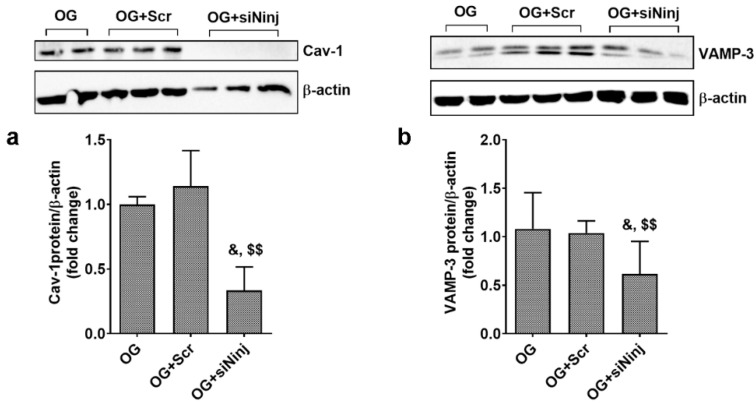
Ninjurin-1 silencing reduces the expression of Cav-1 and VAMP-3 in ECs exposed to oscillating glucose (OG). Cells were transfected with specific siRNA for Ninj-1 (siNinj) or a scrambled siRNA (Scr). At 24 h after transfection, cells were exposed alternatively to 5 mM/25 mM glucose (OG) at every 3 h, for 72 h. (**a**,**b**) Protein expression of Cav-1 (**a**) and VAMP-3 (**b**) in EC lysates relative to β-actin (representative blot and densitometric analysis). All data are expressed as fold change versus OG and presented as mean ± SD. ^&^ *p* < 0.05 vs. OG; ^$$^ *p* < 0.01 vs. OG + Scr.

The involvement of Ninj-1 in the up-regulation of the proteins involved in tran-sendohelial transport was also evaluated in ECs from EA.hy926 line. The results showed that *NINJ-1* silencing (shown in Figure S2) downregulates the protein expression of Cav-1 (0.39 ± 0.18 for siNinj vs. 1.00 ± 0.06 for OG and 1.07 ± 0.2 for Scr; *p* < 0.05 for siNinj vs. OG and *p* < 0.01 for siNinj vs. Scr) and the expression of VAMP-3 (0.61 ± 0.34 for siNinj vs. 1.00 ± 0.37 for OG and 1.04 ± 0.12 for Scr; *p* < 0.05 for siNinj vs. OG and *p* < 0.01 for siNinj-1 vs. Scr) (Figure 9a,b). 

### 3.9. OG Stimulates the Transendothelial Transport Proteins in Primary EC Partially through Mechanisms Involving Ninj-1

The stimulation of the expression of transendothelial proteins by OG is one of the key findings of this paper. To validate the data obtained in EAhy.926 cells, we used primary ECs. The effect of OG on SR-BI, Cav-1, and VAMP-3 protein expression was evaluated in HUVEC’s total lysate using Western Blot.

**Figure 10:**
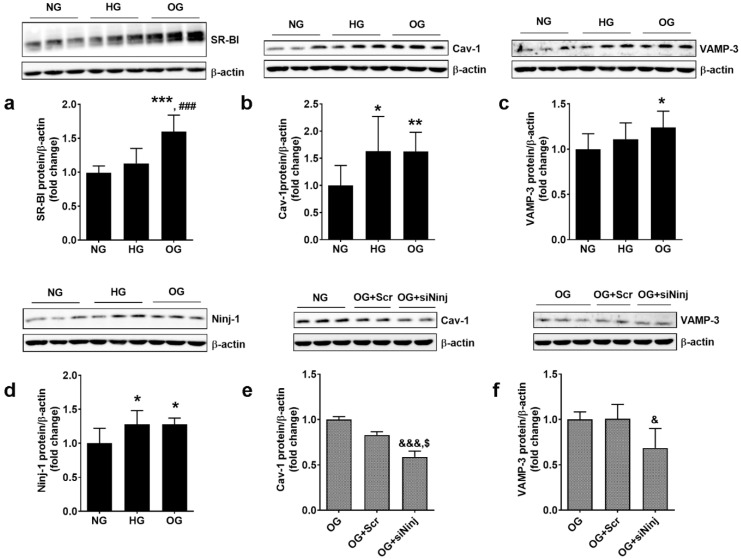
Oscillating glucose stimulates the expression of transendothelial proteins through mechanisms involving Ninjurin-1 in primary ECs (HUVECs). Cells were exposed alternatively to 5 mM/25 mM glucose (OG) at every 3 h, for 72 h (**a**–**d**). In some experiments, 24 h before OG exposure, HUVECs were transfected with specific siRNA for *NINJ-1* (siNinj) or a scrambled siRNA (Scr). At 24 h after transfection, cells were exposed alternatively to 5 mM/25 mM glucose (OG) at every 3 h, for 72 h (**e**,**f**). Protein expression of SR-B1 (**a**), Cav-1 (**b**,**e**), VAMP-3 (**c**,**f**) and Ninj-1 (**d**) in cells’ lysates relative to β-actin (representative blot and densitometric analysis). All data are expressed as fold change versus NG (**a**–**d**) or OG (**e**,**f**) and presented as mean ± SD. * *p* < 0.05, ** *p* < 0.01, *** *p* < 0.001 for OG vs. NG; ^&^ *p* < 0.05, ^&&&^ *p* < 0.001 for siNinj vs. OG; ^###^ *p* < 0.001; ^$^ *p* < 0.05 for siNinj vs. OG + Scr.

In primary HUVECs, the OG exposure determined the increase in SR-B1 protein expression as compared to HG or NG (1.60 ± 0.24 for OG, 1.13 ± 0.22 for HG, and 1.00 ± 0.10 for NG; *p* < 0.001 for OG vs. NG and *p* < 0.001 for OG vs. HG; *p* < 0.001 for all studied groups by ANOVA), shown in Figure 10a. CAV-1 protein expression was up-regulated similarly by OG and HG as compared to NG (1.63 ± 0.35 for OG, 1.63 ± 0.63 for HG, and 1.00 ± 0.37 for NG; *p* < 0.01 for OG vs. NG, *p* < 0.05 for HG vs. NG, *p* = ns for OG vs. HG; *p* < 0.05 for all studied groups by ANOVA) (Figure 10b), while statistically significant up-regulation of VAMP-3 protein expression was only observed with OG (1.24 ± 0.18 for OG, 1.11 ± 0.18 for HG, and 1.00 ±0.17 for NG *p* = 0.05 for OG vs. NG, shown in Figure 10c, confirming the results obtained from EA.hy926 cells.

To validate the hypothesis that Ninj-1 is mediating some of the deleterious effects of OG, we evaluated the expression of this pro-inflammatory protein and its involvement in promoting the alteration of transendothelial transport proteins in primary HUVECs. The protein expression of Ninj-1 after OG exposure was determined and the effect of *NINJ-1* silencing on the expression of transendothelial proteins was estimated in the total lysate of HUVECs by Western Blot. The results showed that OG and HG stimulate similarly the protein expression of Ninj-1 (1.28 ± 0.20 for OG, 1.28 ± 0.09 for HG, and 1.00 ± 0.22 for NG; *p* < 0.05 for OG vs. NG, *p* < 0.05 for HG vs. NG and *p* = ns for OG vs. HG; *p* < 0.05 for all studied groups by ANOVA), shown in Figure 10d. *NINJ-1* silencing (shown in Figure S3) determined a statistically significant decrease in Cav-1 protein expression (0.59 ± 0.06 for siNinj1 vs. 1.00 ± 0.04 for OG and 0.89 ± 0.06 for Scr; *p* < 0.01 for siNinj vs. OG and *p* < 0.05 for siNinj vs. Scr) as shown in Figure 10e, similar to the results obtained in EAhy.926 cells. VAMP-3 protein expression was decreased by *NINJ-1* silencing in OG-exposed cells (0.68 ± 0.21 for siNinj vs. 1.00 ± 0.08 for OG and 1.00 ± 0.15 for Scr; *p* < 0.05 for siNinj vs. OG and *p* = 0.07 for siNinj vs. Scr), shown in Figure 10f. 

## 4. Discussion

To our knowledge, our study presented the first analysis of the effects of short-term high glucose oscillations on endothelial Ninj-1, TNFR1, RAGE, Cav-1, SR-B1, and VAMP-3 proteins. Our study shows for the first time that the analyzed proteins are stimulated by OG, in part through ROS-dependent mechanisms (Ninj-1, RAGE, VAMP-3), or through NF-kB (Cav-1), while others (TNFR1 and SR-BI) are not modulated by either, suggesting other mechanisms for their activation. In addition, our data show that Ninj-1 silencing reduces the expression of transendothelial transport proteins (Cav-1, VAMP-3). The novelty of the reported data provide insight to Ninj-1 as a new therapeutic target for the alleviation of EC dysfunction induced by OG.

The fluctuation of the blood glucose concentration applies both to the short-term daily glucose variations (postprandial) and to the long-term variations, measured as changes in fasting plasma glucose concentrations during the visit-to-visit clinical controls [53]. Many studies reporting correlations between the fluctuations of blood glucose concentration and cardiovascular events refer mainly to the long-term variations observed in diabetic patients [5,6,11,12,13,15], and only a small number of studies analyze the effect of diurnal glucose fluctuations [54]. The present study started from existing data regarding the effects of glucose fluctuations in vivo and aims to identify molecular mechanisms and new possible targets, which could ameliorate these deleterious effects.

Endothelial inflammation and low-density lipoproteins (LDL) accumulation in the subendothelium represent two early steps in the inception of atherosclerosis. Many re-ported investigations are focused on the late stages of atherosclerosis in diabetes, which try to diminish the atherosclerotic plaque vulnerability and to limit its rupture and thrombosis because these features determine the morbidity and mortality of the patients. However, the attention given to the early stages of atherosclerosis is crucial because the pathological alterations at these points are reversible.

Ninjurin-1 was first identified as a two-pass membrane protein that contributes to nerve regeneration after an injury [45]. Beyond the nervous system, Ninj-1 is basally ex-pressed on the surface of the cells of the immune system, as well as on EC, and plays a role in mediating cellular adhesion processes by homophilic interaction. Accumulating data demonstrate that Ninj-1 plays important roles in the development of various pathologies including inflammatory processes [55], diabetes [42], atherosclerosis [43], stroke [44], cancer [56], or erectile dysfunction [46]. Here, for the first time, we report that exposure of ECs to OG increases Ninj-1 levels. Wang et al., using gain- and loss-of function experiments, showed that Ninj-1 stimulates the expression of pro-inflammatory genes, such as MCP-1, in ECs exposed to constant HG [42]. In addition, it is known that Ninj-1 participates in the adhesion and transmigration of inflammatory cells into the subendothelium [51,55]. Our present results obtained on ECs from EA.hy926 line demonstrate that OG stimulates the expression of both MCP-1 and Ninj-1, supporting a link between these two proteins. The increase in these proteins is in association with the increased monocyte adhesion to ECs when exposed to OG under these experimental conditions. Our data regarding the effects of OG on monocyte adhesion are in agreement with other previously published studies [57,58,59,60]. However, the mechanisms supporting this process are inconsistently and contradictorily presented [57,58]. The data obtained in the present study reveal a possible new mechanism for the promotion of monocyte adhesion, indicating Ninj-1 as a possible therapeutic target to reduce inflammation produced by high glucose fluctuations.

A key process in the progression of atherosclerosis is the increased transcytosis of LDL and other proteins from plasma into the subendothelium [61]. Transcytosis is a multi-step process that involves successive caveolae budding from the luminal plasmalemma (endocytosis) and translocation across the EC, followed by fusion with the basal plasmalemma (exocytosis). Recent evidence shows that endothelial SR-BI plays an important role in the transendothelial transport by mediating LDL binding and internalization [61]. The major structural component of caveolae is Cav-1, an integral membrane protein, whose depletion was demonstrated to prevent the formation of caveolae and to ablate albumin transcytosis in vivo [62]. VAMP-3 was shown to participate in LDL exocytosis in cultured ECs exposed to C-reactive protein [63]. In the present paper, we show in an EC line and in primary ECs that OG up-regulates Cav-1 protein expression and stimulates the expression of SR-BI and VAMP-3 proteins more than HG. These new data show that short-term exposure of ECs to fluctuating high concentrations of glucose can determine the increase in transcytosis, thus contributing to the development of diabetes accelerated CVD.

Fluctuations of the blood glucose high concentrations were positively associated with the oxidative stress in diabetic patients [64]. Given the importance of oxidative stress in the stimulation of inflammation and transendothelial transport proteins [63], we were interested in identifying the possible sources of oxidative stress in ECs exposed to short-term OG. Our results demonstrate that the exposure of ECs from EA.hy926 line to OG generates higher levels of ROS compared to constant HG, which is in good agreement with several other studies reporting increased ROS levels in ECs subjected to OG for longer time-periods: 6 h [41], 12 h [38], 16 h [65], or even 24 h [66]. All these data together with ours indicate that the oxidative stress is generated by the oscillations of glucose concentration, independent of their time width. To identify the molecular mechanisms of oxidative stress induction, different possible ROS sources were investigated: the pro-oxidant VPO1 and p22phox, the regulatory subunit of NADPHox, and the antioxidant HO-1. The obtained results show that VPO1 and p22phox are upregulated by OG more than by HG, while HO-1 expression is similar for OG and HG. The endoplasmic reticulum (ER) is the key organelle in the synthesis of proteins and lipids. When ER homeostasis is perturbed, an accumulation of misfolded and unfolded proteins in the ER appears, generating ER stress (ERS). Existing data show that ERS is accompanied by ROS increase, the folding and maturation of proteins being correlated with the depletion of intracellular antioxidants [67]. Our results show that OG stimulates the ERS sensors (GRP78, p-eIF2α, and sXBP1) similarly to HG in EC; these data add to the study of Maamoun et al. [65]. All these data suggest that VPO1 and NADPH oxidase contribute to the up-regulation of ROS in OG-exposed ECs to a greater extent than in HG-exposed ECs, while ERS contributes equally to ROS in OG- and HG-exposed EC, and the antioxidant protection remains the same in the two conditions.

A well-known consequence of ROS production is the activation of the pro-inflammatory signaling pathways including NF-kB and p38 MAPK. Interestingly, in agreement with the increased ROS production, OG stimulates the nuclear translocation of NF-kB in ECs from EA.hy926 line more than HG, while p38 MAPK was equally stimulated by both OG and HG. Using specific inhibitors for ROS and NF-kB transcription factor, we show for the first time that OG stimulates Ninj-1 protein in a ROS-dependent manner, while MCP-1 is regulated by NF-kB. The involvement of ROS in Ninj-1 regulation is in good agreement with the data obtained before by our group in ECs exposed to TNFα, suggesting that the regulation of Ninj-1 by oxidative stress is a universal mechanism, independent of the stimulus or of the experimental model [51]. As in the case of Ninj-1 inhibition, the monocyte adhesion was alleviated by the anti-oxidant NAC, confirming the possible involvement of Ninj-1 in monocyte adhesion in these experimental conditions.

A crucial role in the advancement of ECD and the appearance of cardiovascular complications is played by the pro-inflammatory receptors TNFR1 and RAGE [37,68,69]. Our results demonstrate for the first time that OG stimulates TNFR1 and RAGE expression more compared with HG in EC. We report that RAGE upregulation is determined by the activation of NF-kB induced by OG, confirming other studies showing that the gene of RAGE contains putative NF-kB-like binding sites in the promoter region [70]. In addition to NF-kB, the oxidative stress seems to play an important role in the stimulation of RAGE in OG-exposed cells, since incubation with NAC reduces the expression of this receptor. It is well known that in pro-diabetic conditions, the interaction between RAGE and its ligands determines the production of ROS, NF-kB translocation, and inflammation in ECs [37,68,69]. Interestingly, our data demonstrate that NF-kB activation promotes the transcription of RAGE in OG-exposed EC, thus generating a self-sustained activation cycle and possibly exacerbating the inflammatory stress observed in vivo [70].

Regarding the up-regulation of the transendothelial transport proteins, our data show for the first time that OG stimulates VAMP-3 in a ROS-dependent manner, while Cav-1 is up-regulated by NF-kB activation, in good agreement with Deregowski et al., who indicate that Cav-1 is one of the NF-kB target genes [71]. Our data showing that VAMP-3 and Cav-1 are stimulated by ROS and NF-kB and those of Wang et al. reporting that the functional blocking of Ninj-1 inhibits ROS and NF-kB [42] allowed us to hypothesize that the inhibition of Ninj-1 decreases the expression of the transendothelial transport proteins. In our experiments, Ninj-1 silencing reduced the expression of Cav-1 and, to a lesser extent of VAMP-3, the proteins greatly involved in LDL transcytosis, indicating Ninj-1 as a promising therapeutic target for the downregulation of the transendothelial transport, and thus for the reduction of the atherosclerotic plaque evolution.

**Figure 11:**
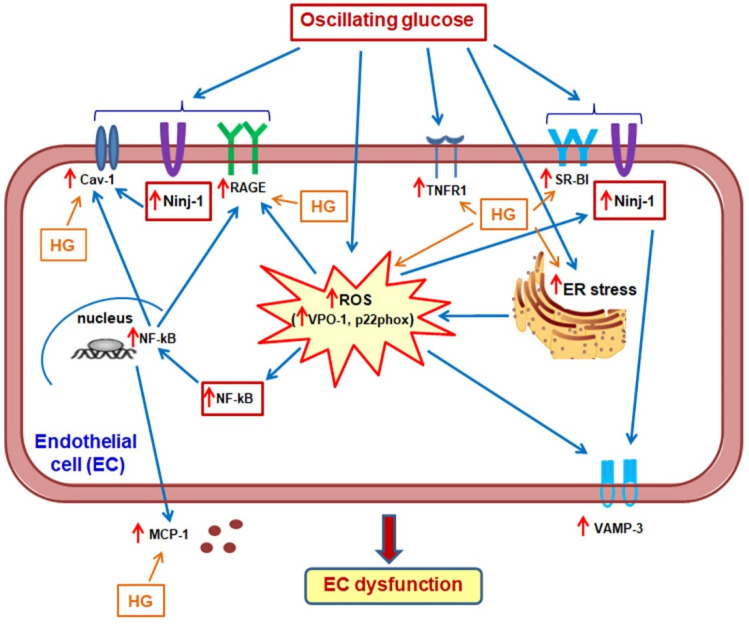
Proposed mechanisms by which oscillating glucose (OG) generates EC dysfunction (ECD). OG determines advanced ECD through a complex process: (1) it enhances intracellular inflammatory stress, determining an increase of Ninj-1, MCP-1 and monocyte adhesion; (2) it stimulates the expression of RAGE and TNFR1; (3) it promotes the up-regulation of SR-BI and VAMP-3, the proteins involved in LDL transendothelial transport, an important process in atheroma formation. These processes are stimulated through mechanisms dependent on the increase of oxidative stress (through VPO1 and p22phox stimulation) and the nuclear translocation of NF-kB. Importantly, Ninj-1 seems to play an intermediate role between inflammation and transendothelial transport.

It is well known that glucose concentrations rise rapidly in the human’s plasma after a meal, the postprandial glucose profile being highly dependent on the carbohydrate ingestion, glucagon regulation, and insulin secretion and/or signaling [72]. In normal subjects, the glucose concentration returns to preprandial levels within 2–3 h [73]. However, in diabetic subjects due to defective insulin signaling, the postprandial excursions are higher, more prolonged, and more challenging to control [72]. In the present manuscript, we used an in vitro model, which mimics the increase in postprandial glucose after the ingestion of three daily meals, by simply exposing the ECs for 3 h to 25 mM glucose, followed by their incubation in 5 mM glucose for another 3 h. The use of the in vitro model may represent a limitation of our study, but the simplicity of the in vitro models makes them helpful in the attempt to understand part of the molecular mechanism representative for the in vivo systems. EA.hy926, the cells that were mostly used in this study, are a widely accepted model in the literature, being used for the identification of different molecular mechanisms, including those related to inflammatory stress or oxidative stress [74,75,76]. EA.hy926 cell line was also used successfully to evaluate the deleterious effects of diabetic conditions [77,78]. The same EC line was recently used with success by our group to demonstrate that hyperglycemia changes the functional phenotype of high-density lipoproteins from diabetic patients with peripheral artery disease towards a pro-inflammatory phenotype [79]. In the present study, the stimulatory effect of OG on the transendothelial transport proteins and the modulatory role of Ninj-1 in the upregulation of Cav-1 and VAMP-3 was validated in EA.hy926 cells, as well as in primary HUVECs. In summary, the present study demonstrates that short-term exposure of ECs to OG induces a complex process involving the stimulation of pro-inflammatory molecules (Ninj-1, MCP-1), cellular receptors (RAGE and TNFR1), and transendothelial transport proteins (SR-BI, Cav-1 and VAMP-3). The increase in oxidative stress through VPO1 and p22phox stimulation and the increase in the nuclear translocation of NF-kB are at the base of these changes. An important role in the stimulation of the transendothelial transport proteins expression is played by Ninj-1, its silencing determining the reduction of Cav-1 and VAMP-3 expression (Figure 11).

We showed that OG determines EC dysfunction, indicating the inflammatory stress as an important player in OG-promoted deleterious effects. Based on the obtained results, we assume that the reduction in inflammatory stress, of Ninj-1 in particular, together with the reduction in postprandial glucose by controlling the carbohydrate intake, food composition, physical exercise, and the use of medication such as sodium glucose transporter 2 inhibitors or glucagon-like peptide 1 agonists (considered safe compounds, able to reduce the postprandial hyperglycaemia with a low risk of hypoglycaemic events) in diabetic patients [72] could diminish the deleterious effects of postprandial hyperglycaemia and glucose oscillations.

We may assume that molecules known to stimulate oxidative stress and NF-kB can induce endothelial dysfunction through similar effects. In the context of diabetes, it would be of interest to investigate whether: (1) pro-inflammatory stimuli (such as TNFα or LPS) known to stimulate Ninj-1 expression and oxidative stress [55]; (2) AGE products as ligands for RAGE and stimulators of oxidative and inflammatory stress [37,52]; (3) different DAMPs (including HMGB-1) known to increase the inflammatory stress and the transendothelial transport [80] can activate similar pathways as the ones in the present manuscript. Importantly, it is of interest in the future to investigate to what extent the concept of “metabolic memory”, a process by which cells can remember episodes of HG-exposure and later induce cardiovascular complications despite a good glycaemic control [81], applied to EC, could involve mechanisms triggered by OG. In addition, the study of OG effects after the long-term HG stimulation is an interesting and important perspective for understanding in depth the effects of glucose variations in diabetic patients.

## 5. Conclusions

It is documented that for the prevention of major adverse cardiovascular events in diabetic patients, a strategy based on a multifactorial, intensive treatment of the cardiovascular risk factors is needed [82]. Starting from this observation, in this study, we aimed to identify new molecular mechanisms and possible therapeutic targets which, together with the existing therapies for alleviating glucose oscillations, could help to reduce the vascular complications that appear in diabetic patients. Based on the results reported in this manuscript, we conclude that short-term fluctuation of high glucose that mimics the post-prandial hyperglycaemia in diabetic patients can aggravate atherosclerosis by determining advanced ECD manifested as exacerbated inflammatory and oxidative stress and increased transendothelial transport. We propose that Ninj-1 plays a regulatory role between inflammation and transendothelial transport, making this protein an attractive therapeutic target for the alleviation of the processes involved in diabetes accelerated atherosclerosis.

## Data Availability

Data is contained within the article or supplementary material.

## Supplementary Materials

The following supporting information can be downloaded at: https://www.mdpi.com/article/10.3390/biom13040626/s1, Figure S1: Oscillating glucose more than constant high glucose increases p22phox in EC; Figure S2: Ninj-1 gene expression levels after specific silencing, in ECs exposed to OG; Figure S3: Ninj-1 protein expression levels after specific silencing, in primary HUVECs exposed to oscillating glucose; Table S1: Primer sequences used for gene expression analysis by Real-Time PCR.
