# Supplementary material for: Oscillating Glucose Induces the Increase in Inflammatory Stress through Ninjurin-1 Up-Regulation and Stimulation of Transport Proteins in Human Endothelial Cells
Source: Biomolecules. 2023 Mar 30;13(4):626. doi: 10.3390/biom13040626 (PMC10135653; doi:10.3390/biom13040626)
Supplement: Supplementary file 1 [file biomolecules-13-00626-s001.zip › biomolecules-2315488-supplementary.pdf]

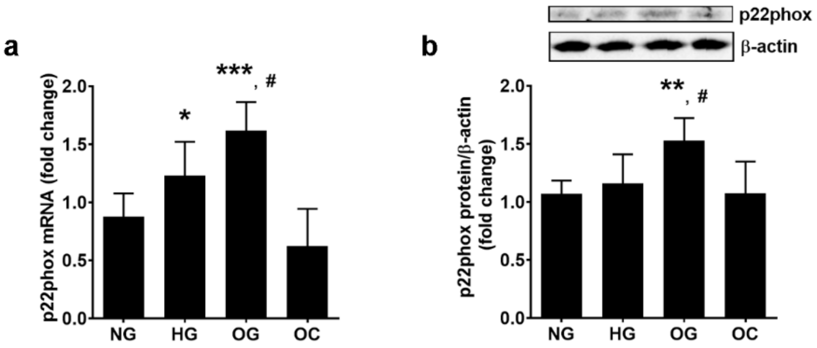

Figure S1. Oscillating glucose more than constant high glucose increases p22phox in EC. Cells were exposed to 5 mM glucose (NG), 25 mM glucose (HG) or alternatively to 5 mM/25 mM glucose (OG) at every 3 h, for 72 h. Cells exposed alternatively to L-glucose or mannitol (using the same protocol as for OG) were considered osmotic control (OC). (a) p22phox mRNA; (b) p22phox protein expression relative to β-actin (representative blot and densitometric analysis). All data are expressed as fold change versus NG and presented as mean ± SD. *p < 0.05, **p < 0.01, ***p < 0.001 vs NG; #p < 0.05 vs HG.

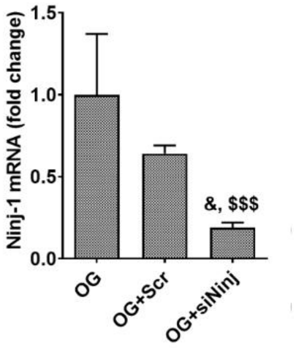

Figure S2. Ninj-1 gene expression levels after specific silencing, in EC from EA.hy926 line exposed to oscillating glucose. Cells were transfected with specific siRNA for Ninj-1 (siNinj) or a scrambled siRNA (Scr). At 24 h after transfection, cells were exposed alternatively to 5 mM/25 mM glucose (OG) at every 3 h, for 72 h. Data are expressed as fold change versus OG and presented as mean ± SD. $^{\&}$p < 0.05 vs OG; $^{\$\$\$}$p < 0.001 vs OG+Scr.

Table S1. Primer sequences used for gene expression analysis by Real-Time PCR

Gene	GeneBank accession number	Sequences of oligonucleotide primers	Amplicon size (bp)
NINJ-1	NM_004148.3	FW: 5'-TCAAGTACGACCTTAACAACCCG-3'<br>RV: 5'-TGAAGATGTTGACTACCACGATG-3'	102
MCP-1	NM_002982.3	FW: 5′-GATCTCAGTGCAGAGGCTCG-3′<br>RV: 5′-TGCTTGTCCAGGTGGTCCAT-3′	153
TNFα	NM_000594.4	FW: 5′-GAGGCCAAGCCCTGGTATG-3′<br>RV: 5′-CGGGCCGATTGATCTCAGC-3′	91
TNFR1/ TNFRSF1A	NM_001065.3	FW: 5′-TCACCGCTTCAGAAAACCACC-3′<br>RV: 5′-GGTCCACTGTGCAAGAAGAGA -3′	96
RAGE/ AGER	NM_001136.4	FW: 5′-CTACCGAGTCCGTGTCTACCA-3′<br>RV: 5′-CATCCAAGTGCCAGCTAAGAG-3′	146
SR-BI/ SCARB	NM_005505.5	FW: 5′-CTGTGGGTGAGATCATGTGG-3′<br>RV: 5′-GCCAGAAGTCAACCTTGCTC-3′	216
CAV-1	NM_001753.4	FW: 5′-AACCTCCTCACAGTTTTCATCCA-3′<br>RV: 5′-GTCGTACACTTGCTTCTCGCTCA-3	165
VAMP-3	NM_004781.4	FW: 5′-GTGGTGGACATAATGCGAGTT-3′<br>RV: 5′-CGTCTGCACGGTCGTCTAAC-3′	82
VPO1/ PXDN	NM_012293.3	FW: 5′-AGCCAGCCATCACCTGGAAC-3′<br>RV: 5′-TTCCGGGCCACACACTCATA-3′	136
GRP78	NM_005347.4	FW: 5′-GAAAGAAGGTTACCCATGCAGT -3′<br>RV: 5′-CAGGCCATAAGCAATAGCAGC -3′	149
sXBP1	NM_005080.3<br>NM_001079539.1	FW: 5′-GCAGGTGCAGGCCCAGT-3′<br>RV: 5′-GAATGCCCAACAGGATATCAGACT -3′	100
p22phox/ CYBA	NM_000101.3	FW: 5′-GTACTTTGGTGCCTACTCCA-3′<br>RV: 5'-CGGCCCGAACATAGTAATTC-3′	167
ACTB	NM_001101.3	FW: 5'-GTCTTCCCCTCCATCGT -3'<br>RV: 5'-CGTCGCCCACATAGGAAT -3'	82

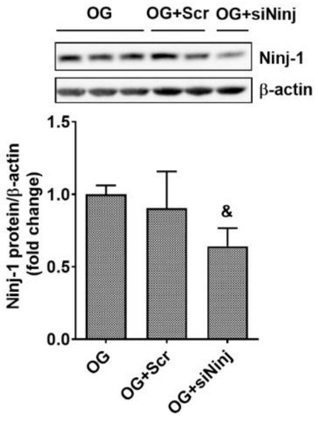

Figure S3. Ninj-1 protein expression levels after specific silencing, in primary HUVECs exposed to oscillating glucose. Cells were transfected with specific siRNA for Ninj-1 (siNinj) or a scrambled siRNA (Scr). At 24 h after transfection, cells were exposed alternatively to 5 mM/25 mM glucose (OG) at every 3 h, for 72 h. Data are expressed as fold change versus OG and presented as mean ± SD. $^{\&}p < 0.05$ vs OG.
